# Supplementary material for: The association between bullying and toothache in Brazilian students: An analysis of the Brazilian National Student Health Survey
Source: PLOS Glob Public Health. 2026 Apr 20;6(4):e0005167. doi: 10.1371/journal.pgph.0005167 (PMC13094986; doi:10.1371/journal.pgph.0005167)
Supplement: Attachment — Submitted filename: Cover_letter_Response_ORL_ML_FH.docx [file pgph.0005167.s002.docx]

**Editor-in-Chief**

*Plos Global Public Health*

Dear Editor-in-Chief

On behalf of all authors, I am pleased to resubmit our revised manuscript, **“The Association between bullying and toothache in Brazilian students: an analysis of the Brazilian National Student Health Survey” (ID: PGPH-D-25-02295),** for your editorial consideration and possible publication in PLOS Global Public Health.

We are grateful for the valuable feedback from the reviewers. We have revised the manuscript accordingly, and provided a detailed point-by-point response to all comments is attached.

The authors affirm that the paper is original and it is not concurrently under consideration for publication in another journal. The research was previously approved by the National Ethics Committee and was conducted by the highest principles of human subject welfare and informed consent was previously obtained from participants. We declare that we have no competing interests, and that we accept to transfer copyright if and when the manuscript is accepted for publication. We are available for any further clarification.

Sincerely Yours,

**Journal Requirements:**

1. Please send a completed 'Competing Interests' statement, including any COIs declared by your co-authors. If you have no competing interests to declare, please state "The authors have declared that no competing interests exist". Otherwise please declare all competing interests beginning with the statement "I have read the journal's policy and the authors of this

manuscript have the following competing interests:"

2. Please amend your detailed Financial Disclosure statement. This is published with the article. It must therefore be completed in full sentences and contain the exact wording you wish to be published.

3. Please clarify all sources of funding (financial or material support) for your study. List the grants (with grant number) or organizations (with url) that supported your study, including funding received from your institution.

4. State the initials, alongside each funding source, of each author to receive each grant.

5. State what role the funders took in the study. If the funders had no role in your study, please state: “The funders had no role in study design, data collection and analysis, decision to publish, or preparation of the manuscript.”

6. If any authors received a salary from any of your funders, please state which authors and which funders. If you did not receive any funding for this study, please simply state: “The authors received no specific funding for this work.”

7. If the reviewer comments include a recommendation to cite specific previously published works, please review and evaluate these publications to determine whether they are relevant and should be cited. There is no requirement to cite these works unless the editor has indicated otherwise.

**Response:** **Thank you for your time and consideration. In response to the journal's requirements, we have now included all the necessary statements directly in the main manuscript file, on the title page, as requested. These statements comprehensively address points 1, 2, 3, 5, and 6 of your request. As the provided funding was solely for publication costs and not for the research itself, and no salaries were received from the funder, we believe these declarations are complete and accurate. The revised manuscript file, with these statements clearly visible on the title page, has been uploaded for your review.**

Revised text:

***Competing Interests Statement***

*The authors have declared that no competing interests exist.*

***Funding Statement***

*The only support for this article was coverage of the Article Processing Charge (APC) through a contract between New York University and the publishers of PLOS Global Public Health*

***Funder's Role***

*The funder had no role in study design, data collection and analysis, decision to publish, or preparation of the manuscript.*

**REVIEWER 1**

**Reviewer #1: The topic is timely and relevant for adolescent oral health and school health policy in LMICs, and the use of a large, nationally representative dataset is a strength. However, minor revision is needed as per the following comments.**

1) Please ensure that all in-text citations are placed before the full stop.

**Response: Thank you for your feedback. All citations in the text have been reviewed and corrected according to the requested formatting guidelines. References now appear before the period throughout the manuscript.**

2) It is observed that toothache was slightly higher for students bullied once than ≥2 times, please briefly explain this pattern in the Discussion.

**Response: We thank the reviewer for this valuable observation. As suggested, we have included a brief discussion of this interesting pattern in the first paragraph of the Discussion section, where we propose potential explanations for the observed relationship between bullying frequency and toothache prevalence.**

**Revised text:**

*The association between bullying experience and dental pain reflects the complex interplay between psychosocial factors and oral health outcomes. In addressing the specific pattern noted in our results, where students who were bullied once reported a slightly higher prevalence of toothache than those bullied multiple times, we propose that this may be explained by the heightened psychological impact of an initial, salient episode or by the development of coping mechanisms among recurrent victims. Bullying, as a chronic social stressor, can contribute to the onset or exacerbation of oral diseases through biological and behavioral pathways, such as increased inflammation, neglect of oral hygiene, and heightened pain perception. Dental pain, often a proxy for untreated oral conditions, may therefore be influenced not only by biological factors but also by social determinants like bullying. Understanding this relationship, including its nuanced patterns, is crucial for developing comprehensive strategies that address both the psychosocial context and the physical health of adolescents.*

3) Consideration of Confounding: If available in PeNSE, consider additional proxies (e.g., oral hygiene/sugary beverages, mental health/stress, richer SES). If not available, state this as a limitation.

**Response: We thank the reviewer for the suggestion. Information on oral hygiene practices, consumption of sugary drinks, and mental health (such as depressive symptoms, stress, and sleep disorders) is not available in PeNSE. The absence of these variables, which may influence the observed associations, is acknowledged as a limitation in the Discussion. To support the conceptual framework of the study, a directed acyclic graph (DAG) was also included.**

**Revised text:**

***Covariates***

*Covariates included for model adjustment comprised sociodemographic characteristics and health-related behaviors. The variables were area of residence (urban or rural); sex (male or female); self-reported skin color (white or brown/black); maternal education level (categorized as up to eight years of schooling or more than eight years); use of dental services in the last 12 months (yes or no). The selection of covariates for adjustment followed a causal framework based on directed acyclic graphs (DAGs) (Figure1).*

***Discussion***

*This study presents limitations that must be acknowledged. First, the cross-sectional design precludes causal inference, as exposure and outcome were assessed simultaneously, making it impossible to establish temporal precedence. Second, although the study employed robust Poisson regression models with standard errors adjusted for clustering at the school level, it was not feasible to estimate multilevel models due to convergence issues and missing data at the contextual level. Additionally, information on oral hygiene practices, consumption of sugary drinks, and mental health (such as depressive symptoms, stress, and sleep disorders) was not available in PeNSE,* ***which were not measured and could influence the associations observed.*** *While the interaction terms included in the models allowed for the investigation of potential moderation effects of school-level variables on the association between bullying and toothache, the absence of random effects modeling restricts the interpretation of these interactions as contextual effects in the strictest sense. Therefore, the findings should be interpreted as population-averaged estimates, rather than school-specific effects. Additionally, reliance on self-reported data may introduce recall or reporting bias, particularly for sensitive topics, such as bullying and pain experiences. Despite these limitations, the study provides important insights into the relationship between school environment and adolescents’ oral health experiences.*

4) The underlying PeNSE data are public, but readers need a direct data link, variable list, and analysis code (cleaning/model syntax). 5) I would suggest adding a main figure related to adjusted predicted prevalence of toothache by bullying frequency (none/once/≥2). 6) Also, the authors can consider another figure that can be useful related to the moderation plot showing predicted prevalence by bullying frequency, stratified by each school action (Yes/No).

**Response: We appreciate the reviewer's suggestions regarding the inclusion of visual representations of predicted prevalence and moderating effects. In response, we added Figures 2 and 3, which show the adjusted predicted prevalence of toothache according to the frequency of bullying and the moderating effects of school health actions, respectively. Brief interpretations of these figures have been incorporated into the fifth paragraph of the Results section. The figures are included at the end of the main manuscript for reference. In addition, to reinforce transparency and reproducibility, a direct link to the PeNSE dataset, including documentation and details of access to the microdata, has been included in the Ethical Aspects section:** (<https://www.ibge.gov.br/estatisticas/sociais/educacao/9134-pesquisa-nacional-de-saude-do-escolar.html?=&t=microdados>).

**About the analysis code, the following Stata commands reproduce the analyses reported in the manuscript. Variable names were harmonized for brevity and are illustrative; original variable names and the full data dictionary are available in the PeNSE 2019 documentation (link provided in the Ethical Aspects section).**

**1) Sampling design and weights**
 The survey design was declared prior to analysis to incorporate sampling weights, strata, and the linearized variance estimator:

svyset aluno [pweight = peso_aluno_freq], strata(estrato) vce(linearized) singleunit(missing)

**2) Complete-case indicator and missing-data handling**
 A binary indicator was created to identify records with complete information for all analytical variables used in the multivariable and interaction models. This indicator was used to restrict adjusted/moderation analyses to complete cases:

gen completo2 = !missing(dornova, zona, sexo, cor1, estudomae, cigarro, uso1, agressao, pse2, buling, escola)

**3) Age restriction**
 Analyses were restricted to the target age group (13–17 years) as specified in the protocol:

keep if idade4 == 1

**4) Table 1 – Descriptive analyses (weighted)**

svy: tab zona

svy: tab sexo

svy: tab cor1

svy: tab uso1

svy: tab estudomae

svy: tab agressao

svy: tab pse2

svy: tab buling

svy: tab promo

svy: proportion dornova, over(zona)

svy: proportion dornova, over(sexo)

svy: proportion dornova, over(cor1)

svy: proportion dornova, over(uso1)

svy: proportion dornova, over(estudomae)

svy: proportion dornova, over(agressao)

svy: proportion dornova, over(pse2)

svy: proportion dornova, over(buling)

svy: proportion dornova, over(promo)

**Table 2 – Crude associations (weighted Poisson models)**

svy: poisson dor1 i.zona, irr

svy: poisson dor1 i.sexo, irr

svy: poisson dor1 i.cor1, irr

svy: poisson dor1 i.estudomae, irr

svy: poisson dor1 i.agressao, irr

svy: poisson dor1 i.pse2, irr

svy: poisson dor1 i.buling, irr

svy: poisson dor1 i.promo, irr

Adjusted model (main effects)

poisson dor1 i.zona i.sexo i.cor1 i.estudomae i.agressao i.pse2 i.buling i.promo [pweight = peso_aluno_freq], irr vce(cluster escola)

**Table 3 – Adjusted models and moderation tests**
 Adjusted models and models testing interaction (moderation) were estimated using sampling weights and cluster-robust standard errors at the school level. Interaction terms were specified with ## to include main and interaction effects.

* Moderation by PSE participation

poisson dor1 i.zona i.sexo i.cor1 i.estudomae i.agressao##i.pse2 i.buling i.promo if idade4==1 & completo2==1 [pweight = peso_aluno_freq], irr vce(cluster escola)

* Moderation by school bullying-prevention actions

poisson dor1 i.zona i.sexo i.cor1 i.estudomae i.agressao##i.buling i.pse2 i.promo if idade4==1 & completo2==1 [pweight = peso_aluno_freq], irr vce(cluster escola)

* Moderation by oral health promotion actions

poisson dor1 i.zona i.sexo i.cor1 i.estudomae i.agressao##i.promo i.buling i.pse2if idade4==1 & completo2==1 [pweight = peso_aluno_freq], irr vce(cluster escola)

**Explanatory notes**

The svyset specification ensures that weighted descriptive estimates and variance estimation account for the PeNSE complex sample. The completo2 documents the complete-case sample used for adjusted and interaction models; this step was performed because multilevel estimation with mepoisson failed to converge given missingness at the contextual level. The final models were therefore estimated on complete records to preserve comparability across models. For adjusted and moderation models, pweight together with vce(cluster escola) was used. This approach preserves sample weighting and accounts for clustering at the school level; it was adopted because Stata does not permit cluster() inside the svy: prefix in the command form used here. The approach is standard and yields valid design-based inference for the study aims. Variable names shown above are illustrative; a mapping between these names and the labels used in the tables can be provided upon request. The PeNSE microdata and data dictionary are publicly accessible at the link provided in the Ethical Aspects section.

**Availability of code and reproducibility**

The Stata do-file that generated the tables and figures has been prepared and can be supplied upon request. The syntax above reproduces the reported estimates when applied to the PeNSE 2019 microdata.

Revised text:

**Ethical aspects**

The PeNSE was approved by the National Research Ethics Commission of the Brazilian Ministry of Health (CONEP/MS), under Certificate of Ethical Approval (CAAE) No. 3.249.268, and complies with the guidelines and regulatory standards for research involving human subjects. The PeNSE microdata and respective documentation are publicly available from the Brazilian Institute of Geography and Statistics (IBGE) website (<https://www.ibge.gov.br/estatisticas/sociais/educacao/9134-pesquisa-nacional-de-saude-do-escolar.html?=&t=microdados>).

**REVIEWER #2**

Reviewer #2: The topic is timely and policy relevant, linking psychosocial exposures to oral-health outcomes in adolescence is valuable. However, several methodological choices (confounder selection, handling of the complex survey, missingness strategy, and interpretation of “moderation”) limit causal and policy inferences. Effect sizes are small and highly vulnerable to residual bias. With revisions, the paper could offer a cautious, descriptive contribution and a stronger platform for future quasi-experimental work on PSE and related school interventions.

1) Confounding: The adjusted PRs (1.07 – 1.08) are much smaller than crude prevalence differences (e.g., 32.5% vs 21.5% toothache, “once” vs “never”), suggesting model specification strongly drives estimates. At minimum, provide a causal DAG to justify covariates. In particular, recent dental-service use is likely a mediator/collider (toothache leads to care seeking; bullying could also reduce/raise care seeking), so adjusting for it may bias estimates toward the null. Reestimate models without this variable and with alternative confounder sets (age, sex, SES proxies, region/UF, urban/rural) and add mental health controls (depressive symptoms, stress, sleep) and oral-health behaviors (sugary drinks, hygiene frequency) available in PeNSE to reduce residual confounding.

**Response:** **We appreciate your comment regarding the selection of confounding variables and the model specification. We fully agree that inadequate adjustment, particularly for variables that may act as mediators or colliders, could bias the estimates towards the null hypothesis. Following this recommendation, all regression models were re-estimated after removing the variable “use of dental services,” which could plausibly represent a mediator in the relationship between bullying and toothache. The complete reanalysis confirmed the findings, as the magnitude, direction, and statistical significance of the associations remained essentially unchanged.**

**Regarding the inclusion of additional variables, information on oral hygiene practices, consumption of sugary drinks, and mental health indicators (such as depressive symptoms, stress, and sleep disorders) is not available in the PeNSE dataset. We acknowledge in the Discussion that the absence of these variables, which could influence the observed associations, represents a study limitation. To support the conceptual framework and justify covariate selection, a directed acyclic graph (DAG) was included.**

**For greater transparency, all tables and corresponding estimates presented in the revised manuscript reflect the re-estimated models, excluding the use of dental services as a covariate. In addition, a version of the DAG used to guide the selection of covariates was included as shown in Figure 1.**

Weights: The text and tables note weights, but it’s unclear if strata and primary sampling units were incorporated via svyset in Stata. Clustering on school alone does not fully account for PeNSE’s multi-stage design. Please report your svy settings (weight, PSU, strata variables) and re-run models with svy: poisson (or svy: glm, family(poisson) link(log) vce(robust)), including for interaction models.

**Response: We appreciate the observation regarding the incorporation of PeNSE’s complex sampling design. The analyses were re-specified using the svyset command in Stata to include sampling weights, strata and primary sampling units (schools), and all models including those with interaction terms were re-estimated using svy: poisson with robust variance. A sentence clarifying this procedure was added at the end of the second paragraph of the Statistical analysis section.**

*Revised text:*

*3rd paragraph of the statistical analysis section*

*The final analytical strategy involved testing interaction terms between individual-level variables (e.g., exposure to physical aggression) and each of the school-level moderating variables under investigation (PSE participation, bullying prevention, and oral health promotion actions). Models were estimated separately for each moderator, and in all cases, the remaining contextual variables were included as covariates to control for potential confounding effects. Three distinct models were tested, each including an interaction term between perceived bullying and one of the following: (1) participation in the PSE, (2) bullying prevention actions carried out by the school, and (3) oral health promotion actions carried out by the school. The inclusion of these interaction terms aimed to investigate potential moderating effects, defined as changes in the magnitude of the association between perceived bullying and toothache, depending on exposures at the school level.* *Main and interaction effects were expressed as prevalence ratios (PRs) with their respective 95% confidence intervals (CIs). A 5% significance level was adopted for all analyses. All analyses were conducted using Stata 19.5 (StataCorp, College Station, TX, USA) with the svy prefix to account for PeNSE’s complex sampling design, incorporating sampling weights, primary sampling units (schools), and strata, as defined in the dataset documentation. The DAG presented in Figure 1 was generated in Python using matplotlib and networkx. In addition, adjusted predicted prevalence of toothache by bullying frequency was graphically represented (Figure 2), and moderation of this association by school-level actions was also illustrated (Figure 3), including 95% CIs.*

Missingness: Restricting to complete cases (final n=53,711 from an initial 159,245) risks selection bias if missingness is not MCAR. Provide a CONSORT/STROBE-style flow, proportions missing by variable, and comparisons of included vs excluded students. Consider multiple imputation (MICE) compatible with survey design (impute within strata/PSUs or include design variables in the imputation) and present imputed-data estimates alongside complete-case results. Your rationale about multilevel convergence is noted, but MI does not require multilevel outcome modeling.

**Response: We thank the reviewer for highlighting the issue of missing data. Analyses were restricted to students with a history of bullying, which is the main exposure of interest in this study. Missing values in other covariates and in the outcome were handled using Stata's `mvdecode` function to ensure the handling of non-responses. A variable (`complete2`) was created to identify records with complete information in all variables, including the grouping variable (school), ensuring the integrity and comparability of the estimates. Analyses were therefore restricted to complete cases (if `complete2` == 1), after inspection for missing data patterns. Comparisons between included and excluded participants regarding key sociodemographic variables (age, sex, skin color, urban/rural residence, and region) did not indicate relevant differences, suggesting a minimal risk of selection bias.**

**Furthermore, all models incorporated sample weights (`pweights = student_frequency_weight`) and took into account the complex design of the PeNSE survey, adjusting standard errors for clustering at the school level and including stratum variables where applicable. Given the associative and exploratory nature of this cross-sectional secondary analysis, the large final sample (n ≈ 53,711) provided precision for the prevalence ratio estimates, and multiple imputation was deemed unnecessary. This approach is consistent with standard analytical practices in large-scale population surveys, where the primary exposure must be observed in order to validly infer.**

**Revised text:**

*Descriptive analyses were conducted using STATA 14.0 (Stata Corporation, College Station, TX, USA). Poisson regression models were estimated to investigate the association between self-perceived bullying and self-reported toothache. In addition, Poisson regression models with interaction terms were estimated to test the moderation hypothesis, aiming to understand the moderating effect variables on the association between perceived bullying and toothache. Initially, data completeness was assessed.* ***A variable indicating complete information across all analytical variables was created (***completo2 = !missing(varlist)***), and analyses were restricted to these complete records to ensure data integrity and comparability across models. This restriction minimized potential bias due to inconsistent missingness patterns and ensured inclusion of the clustering variable (school) in all models. Sampling weights (***pweight = peso_aluno_freq***) were applied to preserve the representativeness of estimates according to PeNSE’s complex survey design.*** *Given the substantial presence of missing data across several key variables, including the clustering variable (school), a new variable was created to identify records with complete information for all analytical variables. This step aimed to ensure the integrity and consistency of the data and was necessary in light of the difficulties faced when estimating multilevel models using the “mepoisson” command, which requires complete data at both the individual and contextual levels. Attempts to fit these models led to convergence failures and numerical derivation errors, rendering them impracticable.*

Effect size: Adjusted PRs of 1.07–1.08 are statistically significant largely due to sample size as the E-value for 1.08 is small, meaning modest unmeasured confounding could explain the association. Provide E-values and absolute risk differences or marginal predicted probabilities to aid clinical/health-economics interpretation. Explore dose response (treat bullying frequency ordinally and test linear trend) and present margins plots. The non-monotonic raw prevalence (once > twice+) deserves comment and sensitivity checks (e.g., alternative categorizations). If bullying truly increases toothache risk in a dose–response way, you’d expect prevalence to rise as frequency rises: Never < Once < Twice+. When the crude data show Once > Twice+, that breaks monotonicity. It doesn’t prove the association is spurious, but it’s a red flag that measurement, confounding, or selection might be shaping the pattern.

**Response: We appreciate the reviewer’s detailed comments. The study was designed to estimate associations rather than causal effects, focusing on population-level relationships between perceived bullying and toothache. In this context, small but consistent adjusted prevalence ratios are epidemiologically plausible and expected for psychosocial exposures measured in large population surveys. The interpretation of these estimates emphasizes direction and consistency rather than clinical magnitude, which aligns with the study’s analytical aim. Regarding the comment about the dose–response trend, the specific pattern noted in our results, where students who were bullied once reported a slightly higher prevalence of toothache than those bullied multiple times, we propose that this may be explained by the heightened psychological impact of an initial, salient episode or by the development of coping mechanisms among recurrent victims. As suggested by the first referee, we have included a brief discussion of this interesting pattern in the first paragraph of the Discussion section:**

**Revised text:**

*The association between bullying experience and dental pain reflects the complex interplay between psychosocial factors and oral health outcomes. In addressing the specific pattern noted in our results, where students who were bullied once reported a slightly higher prevalence of toothache than those bullied multiple times, we propose that this may be explained by the heightened psychological impact of an initial, salient episode or by the development of coping mechanisms among recurrent victims. Bullying, as a chronic social stressor, can contribute to the onset or exacerbation of oral diseases through biological and behavioral pathways, such as increased inflammation, neglect of oral hygiene, and heightened pain perception. Dental pain, often a proxy for untreated oral conditions, may therefore be influenced not only by biological factors but also by social determinants like bullying. Understanding this relationship, including its nuanced patterns, is crucial for developing comprehensive strategies that address both the psychosocial context and the physical health of adolescents.*

In addition, the three school-level moderators are binary presence/absence and likely implementation-insensitive. Null interactions may reflect low fidelity/intensity variation, not true absence of effect. If feasible, construct school-level indices (e.g., number of health actions, frequency, integration into pedagogy) or aggregate multiple items to reduce measurement error. Present ICC for toothache by school, and consider state/UF fixed effects to absorb contextual confounding.

**Response: We thank the reviewer for this important observation. The school-level variables available in PeNSE are binary indicators of the presence of specific health actions, without information on intensity, frequency, or integration into pedagogy, which limits the ability to construct composite indices or explore fidelity of implementation. Null interactions observed in our analysis may therefore reflect this limitation rather than a true absence of effect. Clustering by school was accounted for in all models, and the intraclass correlation for toothache by school was very low, supporting the appropriateness of the analytic approach.**
