# Supplementary material for: The association between bullying and toothache in Brazilian students: An analysis of the Brazilian National Student Health Survey
Source: PLOS Glob Public Health. 2026 Apr 20;6(4):e0005167. doi: 10.1371/journal.pgph.0005167 (PMC13094986; doi:10.1371/journal.pgph.0005167)
Supplement: Attachment — Submitted filename: R2_Response-2_.docx [file pgph.0005167.s003.docx]

Journal Requirements:

If the reviewer comments include a recommendation to cite specific previously published works, please review and evaluate these publications to determine whether they are relevant and should be cited. There is no requirement to cite these works unless the editor has indicated otherwise.

Please review your reference list to ensure that it is complete and correct. If you have cited papers that have been retracted, please include the rationale for doing so in the manuscript text, or remove these references and replace them with relevant current references. Any changes to the reference list should be mentioned in the rebuttal letter that accompanies your revised manuscript. If you need to cite a retracted article, indicate the article’s retracted status in the References list and also include a citation and full reference for the retraction notice.

Additional Editor Comments (if provided):

**Response:** **Dear Editor, on behalf of all authors, we thank you for the opportunity to further revise our manuscript. We are grateful for the careful evaluation and constructive comments provided by the reviewers. We have carefully addressed all remaining points raised during this second round of review, with particular attention to clarifying the analytical strategy for the complex survey design and improving transparency regarding missing data and sample selection. All changes are detailed below and have been incorporated into the revised manuscript and supplementary material.**

**Review Comments to the Author**

Please use the space provided to explain your answers to the questions above. You may also include additional comments for the author, including concerns about dual publication, research ethics, or publication ethics. (Please upload your review as an attachment if it exceeds 20,000 characters)

**Reviewer #1:** The author has adequately responded to all comments and queries, providing substantial revisions that have significantly improved the clarity and rigor of the work.

**Response: We sincerely thank Reviewer 1 for the positive and encouraging evaluation of the revised manuscript. We appreciate the recognition that the revisions improved the clarity and rigor of the study.**

**Reviewer #2:** Thank you for the revision and detailed response to Round 1. The manuscript is meaningfully improved and several core methodological concerns have been addressed, notably: (i) the inclusion of a causal framework (DAG) clarifying which variables are treated as confounders versus potential mediators, (ii) corresponding adjustments to the covariate strategy to avoid overadjustment, and (iii) the addition of predicted/adjusted prevalence figures that substantially improve interpretability relative to reporting only prevalence ratios.

That said, I still see one major issue and a few important transparency items that should be resolved before the paper is fully publication-ready:

Major: internal inconsistency in the stated estimation approach for the complex survey design. The revised methods indicate analyses were conducted using Stata’s survey procedures with svy and incorporation of weights/PSUs/strata, but the Statistical Analysis section also discusses the use of robust variance estimation with standard errors clustered at the school level as an “alternative strategy.” These are not equivalent approaches, and the manuscript currently reads as if both are used without a clear statement of which is the primary specification and which (if any) is a sensitivity check. This needs to be made internally consistent: either (a) present the survey-design-based estimation as the main approach and remove/relegate the cluster-robust discussion to sensitivity analysis, or (b) if survey estimation was not ultimately used, revise the text accordingly and justify the final choice.

**Response: We thank the reviewer for this valuable comment. We agree that the initial version of the manuscript did not clearly describe the analytical strategy adopted for handling the complex sampling design of PeNSE across different stages of the analysis.**

**We have revised the Statistical Analysis section to explicitly clarify that survey procedures using the svy prefix were applied in the descriptive analyses to account for sampling weights, primary sampling units, and strata. For the regression analyses, Poisson models were estimated using sampling weights and robust standard errors clustered at the school level, which constituted the primary analytical specification.**

**Full survey-design–based regression models using the svy prefix were explored during model development. However, due to technical constraints related to missing contextual identifiers and instability in models including interaction terms, this approach was not retained in the final analyses. The adopted strategy allowed appropriate incorporation of sampling weights while ensuring model convergence and interpretability of interaction effects.**

**The manuscript has been amended to clearly distinguish between descriptive and inferential analytical approaches and to specify that regression estimates should be interpreted as population-averaged associations. We appreciate the reviewer’s comment, which improved the transparency and clarity of the analytical description.**

***3rd paragraph of the statistical analysis section***

**Revised text:**

*The final analytical strategy involved testing interaction terms between individual-level variables (e.g., exposure to physical aggression) and each of the school-level moderating variables under investigation (PSE participation, bullying prevention, and oral health promotion actions). Models were estimated separately for each moderator, and in all cases, the remaining contextual variables were included as covariates to control for potential confounding effects. Three distinct models were tested, each including an interaction term between perceived bullying and one of the following: (1) participation in the PSE, (2) bullying prevention actions carried out by the school, and (3) oral health promotion actions carried out by the school. The inclusion of these interaction terms aimed to investigate potential moderating effects, defined as changes in the magnitude of the association between perceived bullying and toothache, depending on exposures at the school level. Main and interaction effects were expressed as prevalence ratios (PRs) with their respective 95% confidence intervals (CIs). A 5% significance level was adopted for all analyses. Although PeNSE adopts a complex sampling design, survey procedures using the svy prefix were applied only in the descriptive analyses to account for sampling weights, primary sampling units, and strata. For the regression analyses, Poisson models were estimated using sampling weights and robust standard errors clustered at the school level. Full survey-design–based estimation for the regression models was explored; however, due to technical constraints related to missing contextual identifiers and model stability in models including interaction terms, this approach was not adopted in the final analyses. Therefore, the regression estimates should be interpreted as population-averaged associations.*

Transparency: complete-case restriction remains under-documented. While the revised text provides a clearer narrative rationale for complete-case analysis, it still omits the diagnostic information requested in Round 1: missingness rates by variable, a simple sample flow/attrition summary, and a comparison of included vs excluded observations on key demographics/SES markers. Given the large reduction from the initial sample to the analytic sample, a short appendix table would materially strengthen credibility and allow readers to assess potential selection issues.

**Response: We thank the reviewer for emphasizing the importance of transparency regarding the complete-case restriction.** **We fully agree that additional diagnostic information is necessary to allow readers to assess the potential implications of missing data and sample selection. In response to this comment, we have added Appendix 1, which is provided at the end of this response letter and incorporated into the revised manuscript. Appendix 1 includes four complementary tables that document (i) variable-specific missingness patterns, (ii) the analytical sample flow, (iii) comparisons between included and excluded participants on baseline sociodemographic characteristics, and (iv) clustering diagnostics at the school level, thereby fully characterizing the complete-case analytical strategy.**

**Table 1 presents the pattern and proportion of missing data for each analytical variable among eligible adolescents aged 13–17 years.**

**Table 2 provides a flow summary of the analytical sample, detailing the sequential restrictions from the total PeNSE sample to the final complete-case analytic sample.**

**Table 3 compares included and excluded participants according to baseline sociodemographic characteristics (sex, area of residence, skin color, and maternal education), which are not conceptually downstream of the exposure or outcome, in order to assess the potential for selection bias associated with the complete-case approach.**

**Although some differences reached statistical significance due to the large sample size, the overall distributions were broadly similar between included and excluded participants, suggesting a limited risk of selection bias.**

**In addition, we have explicitly referenced the availability of these diagnostic materials in the first paragraph of the Statistical Analysis section by adding the following sentence: *“Detailed information on missing data patterns and comparisons between included and excluded participants is provided in Appendix 1.”***

Moderation measures remain coarse. The revision more clearly acknowledges the limitations of binary indicators for school actions and helps interpret the null interaction results. However, the analysis would benefit from at least minimal additional reporting (e.g., ICC or other clustering diagnostics, if invoked) and/or clearer framing that the moderator measures capture presence/absence rather than intensity/implementation quality. Overall, I view the paper as substantially improved and close, but I recommend the editor request a targeted revision to (i) fully align and clearly state the survey/variance estimation strategy and (ii) add basic missingness/sample-selection diagnostics (ideally in an appendix). These are feasible changes that would materially improve reproducibility and confidence in the results.

**Response: We appreciate the reviewer’s suggestion. In response, we have strengthened transparency and reproducibility by adding detailed methodological diagnostics, all incorporated in the Methods section and in a new Appendix.**

**Specifically, Appendix 1 now includes: (i) variable-specific missingness rates (Table 1), (ii) a flow diagram of the analytical sample from the full PeNSE dataset to the complete-case sample (Table 2), and (iii) a comparison of included versus excluded participants on key baseline sociodemographic characteristics to assess potential selection bias (Table 3).**

**In addition, we formally assessed clustering at the school level by fitting a null random-intercept model for the outcome. Clustering diagnostics, including the intraclass correlation coefficient (ICC) and likelihood-ratio test, are reported in Appendix Table 4 and summarized in the first paragraph of the Statistical Analysis section. These diagnostics indicated negligible between-school variability, supporting the use of population-averaged Poisson regression models with robust standard errors clustered at the school level.**

**Finally, we clarified in the Methods that school-level moderator variables capture the presence or absence of actions rather than their intensity or implementation quality. No changes were made to the Discussion, as these additions relate to analytical strategy and transparency rather than interpretation of substantive findings.**

**Appendix 1**

**Table 1. Pattern of missing data across analytical variables among eligible adolescents (13–17 years).**

| **Variable** | **Eligible N** | **Missing n** | **Missing %** |
| --- | --- | --- | --- |
| Toothache | 65,073 | 11,362 | 17.5 |
| Bullying | 65,073 | 103 | 0.2 |
| Sex | 65,073 | 107 | 0.2 |
| Skin color | 65,073 | 6,022 | 9.3 |
| Mother’s education | 65,073 | 13,816 | 21.2 |
| Area of residence (urban/rural) | 65,073 | 0 | 0.0 |
| Smoking | 65,073 | 148 | 0.2 |
| Alcohol use | 65,073 | 660 | 1.0 |
| Physical aggression | 65,073 | 544 | 0.8 |
| School Health Program (PSE) | 65,073 | 167 | 0.3 |
| School (cluster variable) | 65,073 | 0 | 0.0 |

Note: Missing values include non-response and invalid answers recoded using mvdecode.

**Table 2. Flow of the analytical sample.**

| **Sample definition** | **n** |
| --- | --- |
| Total PeNSE sample | 81,496 |
| Eligible adolescents (13–17 years) | 65,073 |
| Complete-case analytic sample (idade4 == 1 & completo2 == 1) | 38,781 |

## **Table 3. Comparison between included and excluded participants due to missing data**

Base: total sample before age restriction
N = **81,496**

| **Sex** | **Excluded n (%)** | **Included n (%)** |
| --- | --- | --- |
| Male | 21,340 (50.2) | 18,418 (47.5) |
| Female | 21,188 (49.8) | 20,363 (52.5) |

| **Area** | **Excluded n (%)** | **Included n (%)** |
| --- | --- | --- |
| Urban | 38,774 (90.8) | 36,090 (93.1) |
| Rural | 3,941 (9.2) | 2,691 (6.9) |

| **Skin color** | **Excluded n (%)** | **Included n (%)** |
| --- | --- | --- |
| White | 10,330 (29.5) | 11,862 (30.6) |
| Black | 24,698 (70.5) | 26,919 (69.4) |

| **Mother’s education** | **Excluded n (%)** | **Included n (%)** |
| --- | --- | --- |
| ≤ 8 years | 10,372 (42.3) | 14,985 (38.6) |
| > 8 years | 14,156 (57.7) | 23,796 (61.4) |

**Note:** Comparisons were conducted to assess potential selection bias arising from the complete-case approach. Despite some statistically significant differences due to the large sample size, distributions were broadly similar between included and excluded participants, suggesting minimal risk of selection bias.

**Note:** Comparisons were restricted to baseline sociodemographic characteristics not conceptually downstream of the exposure or outcome, aiming to assess potential selection bias associated with the complete-case approach. Variables related to behaviors, violence, school context, exposure, and outcome were intentionally excluded from this comparison.

**Table 4. Clustering diagnostics for the outcome at the school level**

| **Outcome** | **Cluster level** | **ICC** | **LR test (χ²)** | **p-value** |
| --- | --- | --- | --- | --- |
| Dental pain | School | 0.00001 | 0.01 | 0.47 |

Note: A null random-intercept model was fitted to assess clustering at the school level. The between-school variance was negligible, resulting in an intraclass correlation coefficient close to zero (ICC = 0.00001). The likelihood-ratio test comparing the mixed-effects model with a single-level model was not statistically significant (p = 0.47), indicating no meaningful clustering. Given the absence of contextual variance and convergence instability due to boundary estimation, multilevel models were deemed unnecessary, and marginal models with robust standard errors clustered at the school level were retained.
